# Robust Nonlinear Tracking Control with Exponential Convergence Using Contraction Metrics and Disturbance Estimation

**DOI:** 10.3390/s22134743

**Published:** 2022-06-23

**Authors:** Pan Zhao, Ziyao Guo, Naira Hovakimyan

**Affiliations:** Department of Mechanical Science and Engineering, University of Illinois at Urbana-Champaign, Urbana, IL 61801, USA; ziyaog2@illinois.edu (Z.G.); nhovakim@illinois.edu (N.H.)

**Keywords:** robust control, nonlinear control, uncertain systems, disturbance estimation, robot safety

## Abstract

This paper presents a tracking controller for nonlinear systems with matched uncertainties based on contraction metrics and disturbance estimation that provides exponential convergence guarantees. Within the proposed approach, a disturbance estimator is proposed to estimate the pointwise value of the uncertainties, with a pre-computable estimation error bounds (EEB). The estimated disturbance and the EEB are then incorporated in a robust Riemannian energy condition to compute the control law that guarantees exponential convergence of actual state trajectories to desired ones. Simulation results on aircraft and planar quadrotor systems demonstrate the efficacy of the proposed controller, which yields better tracking performance than existing controllers for both systems.

## 1. Introduction

Robotic systems generally have nonlinear dynamics and are subject to model uncertainties and disturbances. Moreover, many robotic systems are underactuated, i.e., having fewer independent control inputs than degrees of freedom, including fixed-wing aircraft, quadrotors and dynamic walking robots. The design of tracking controllers for underactuated robotic systems is a much more challenging problem compared to that for fully-actuated systems. Recently, the concept of a control contraction metric (CCM) was introduced in [1] to synthesize trajectory tracking controllers for general nonlinear systems, including underactuated ones. The CCM extends contraction theory [2] from analysis to constructive control design, while contraction theory is focused on analyzing nonlinear systems in a differential framework by studying the convergence between pairs of state trajectories toward each other. It was shown in [3] that CCM reduces to conventional sliding and energy-based designs for fully-actuated systems. On the other hand, for underactuated systems, compared to prior approaches based on local linearization [4], the CCM approach leads to a convex optimization problem for controller synthesis and generates controllers that stabilize *every* feasible trajectory in a region, instead of just a single target trajectory that must be known a priori [3].

On the other hand, control design methods to deal with dynamic uncertainties in the deterministic setting can be roughly classified into adaptive and robust approaches. Robust approaches, such as $H_{\infty }$ control [5], μ synthesis [6] and robust/tube model predictive control (MPC) [7,8], usually consider parametric uncertainties or bounded disturbances and aim to find controllers with performance guarantees for the worst case of such uncertainties. The consideration of worst-case scenarios associated with robust approaches often leads to conservative nominal performance. Disturbance–observer (DOB) based control and related methods such as active disturbance rejection control (ADRC) [9] lump all uncertainties that may include parametric uncertainties, unmodeled dynamics and external disturbances, together as a “total disturbance”, estimate it via an observer and then compute control actions to compensate for the estimated disturbance [10] to recover the nominal performance. However, for state-dependent uncertainties, DOB-based control methods usually ignore the dependence of “disturbance” on system states and rely on assumptions on the derivative of the “disturbance” that are difficult to verify for theoretical guarantees [10,11]. Alternatively, adaptive control methods such as model reference adaptive control (MRAC) [12] usually need a parametric structure for the uncertainties, rely on online estimation of the parameters for control law construction and provide asymptotic performance guarantees in most cases. One of the exceptions is $L_{1}$ adaptive control [13] that does not need a parameterization of the uncertainties (similar to DOB-based control) and focuses on transient performance guarantees in terms of uniformly bounded error between the ideal and uncertain systems.

Both robust and adaptive control approaches have been explored in the context of CCM-based control in the presence of uncertainties and disturbances. In particular, adaptive control was combined with CCM to handle nonlinear control-affine systems with both parametric [14] and non-parametric uncertainties [15]. The case of bounded disturbances in CCM-based control was addressed by leveraging input-to-state stability analysis [16] or robust CCM [17,18]. CCM for stochastic systems was developed in [19] to minimize the mean squared tracking error in the presence of stochastic disturbances. Closely relevant to this paper, [15] designed an $L_{1}$ adaptive controller to augment a baseline CCM-based controller to compensate for matched nonlinear non-parametric uncertainties that can depend on both time and states. The authors of [15] proved that transient tracking performance was guaranteed in the sense that the actual state trajectory exponentially converges to a neighborhood or a tube around the desired one. Compared to [15], our approach relies on a disturbance observer that yields an estimation error bound and robust Riemannian energy condition and ensures that the actual state trajectory exponentially converges to the nominal one.

*Statement of Contributions:* We present a tracking controller for nonlinear systems subject to matched uncertainties that can depend on both time and states based on contraction metrics and disturbance estimation. Our controller leverages a disturbance estimator to estimate the pointwise value of the uncertainties, with a pre-computable estimation error bound. The estimated disturbance and the estimation error bound are then incorporated into a robust Riemannian energy condition to compute the control law that guarantees exponential convergence of actual state trajectories to nominal ones. We validate the efficacy of our controller on two simulation examples and demonstrate its advantages over existing controllers.

The idea presented in this paper is leveraged in [20] for safe learning of uncertain dynamics using deep neural networks. Compared to [20], this paper is not relevant to learning and allows the uncertainty to be dependent on both time and states, as opposed to the dependence on states only in [20]. Additionally, this paper includes an additional aircraft example for performance illustration and conducts extensive comparisons with existing adaptive approaches in simulations that are not available in [20].

Notations: Let $R^{n}$, $R^{+}$ and $R^{m\times n}$ denote the *n*-dimensional real vector space, the set of non-negative real numbers and the set of real *m* by *n* matrices, respectively. *I* and 0 denote an identity matrix, and  a zero matrix of compatible dimensions, respectively; ‖·‖ denotes the 2-norm of a vector or a matrix. For a vector *y*, $y_{i}$ denotes its *i*th element. For a matrix-valued function $M:R^{n}\to R^{n\times n}$ and a vector $y\in R^{n}$, $\partial_{y}M\left(x\right)≜\sum_{i=1}^{n}\frac{\partial M(x)}{\partial x_{i}}y_{i}$ denotes the directional derivative of M(x) along *y*. For symmetric matrices *P* and *Q*, P>Q (P≥Q) means P−Q is positive definite (semidefinite). 〈X〉 is the shorthand notation of $X+X^{⊤}$. Finally, ⊖ denotes the Minkowski set difference.

## 2. Problem Statement and Preliminaries

Consider a nonlinear control-affine system with uncertainties

(1) $$\dot{x}\left(t\right)=f\left(x\left(t\right)\right)+B\left(x\left(t\right)\right)\left(u\left(t\right)+d\left(t,x\left(t\right)\right)\right),$$

where $x\left(t\right)\in X\subset R^{n}$ is the state vector, $u\left(t\right)\in U\subset R^{m}$ is the control input vector, $f:R^{n}\to R^{n}$ and $B:R^{n}\to R^{m}$ are known and locally Lipschitz continuous functions, and d(t,x) represents the *matched* model uncertainty that can depend on both time and states. We assume that B(x) has full column rank for any x∈X. Suppose X is a compact set that contains the origin, and  the control constraint set U is defined as $U≜\{u\in R^{m}:\underline{u}\leq u\leq \bar{u}\}$, where $\underline{u},\bar{u}\in R^{m}$ denote the lower and upper bounds of all control channels, respectively. Furthermore, we make the following assumptions on B(x) and d(t,x).

**Assumption** **1.** *There exist known positive constants $L_{B}$, $L_{d}$, $l_{d}$ and $b_{d}$ such that for any x,y∈X and t,τ≥0, the following inequalities hold:*

(2) $$\begin{array}{cc} ∥B(x)-B(y)∥ & \leq L_{B}∥x-y∥, \end{array}$$

(3) $$\begin{array}{cc} ∥d(t,x)-d(\tau ,y)∥ & \leq L_{d}∥x-y∥+l_{d}\left|t-\tau \right|, \end{array}$$

(4) $$\begin{array}{cc} ∥d(t,x)∥ & \leq b_{d}. \end{array}$$

**Remark** **1.** *Assumption 1 indicates that the uncertain function d(t,x) is locally Lipschitz in both t and x with known Lipschitz constants and is uniformly bounded by a known constant in the compact set X.*

In fact, given the local Lipschitz constants $L_{d}$ and $l_{d}$, a uniform bound on d(t,x) in X can always be derived by using Lipschitz continuity properties if the bound on $d(t,x^{*})$ for an arbitrary $x^{*}$ in X and any t≥0 is known. For instance, assuming $\left\|d(t,0)\right\|\leq b_{d}^{0}$, from (3), we have $\left\|d(t,x)\right\|\leq b_{d}^{0}+L_{d}\max_{x\in X}\|x\|$ for any x∈X and g≥0. In practice, some prior knowledge about the actual system and the uncertainty may be leveraged to obtain a tighter bound than the one based on the Lipschitz continuity explained earlier, which is why we directly make an assumption on the uniform bound. With Assumption 1, we will show (in Section 3.3) that the pointwise value of d(t,x(t)) at any time *t* can be estimated with a pre-computable estimation error bound.

For the system in (1), assume we have a nominal state and input trajectory, $x^{⋆}\left(\cdot \right)$ and $u^{⋆}\left(\cdot \right)$, which satisfy the nominal, i.e., uncertainty-free, dynamics:

(5) $${\dot{x}}^{⋆}=f\left(x^{⋆}\right)+B\left(x^{⋆}\right)u^{⋆}.$$

We would like to design a state-feedback controller in the form of

(6) $$u\left(t\right)=k\left(t,x\left(t\right),x^{⋆}\left(t\right)\right)+u^{⋆}\left(t\right),$$

so that the actual state trajectory x(·) exponentially converges to the nominal one $x^{⋆}\left(\cdot \right)$. Our solution is based on CCM and disturbance estimation. Next, we briefly review CCM for uncertainty-free systems.

### Control Contraction Metrics (CCMs)

We first introduce some notations related to Riemannian geometry, most of which are from [1]. A Riemannian metric on $R^{n}$ is a symmetric positive-definite matrix function M(x), smooth in *x*, which defines a “local Euclidean” structure for any two tangent vectors $\delta_{1}$ and $\delta_{2}$ through the inner product ${\langle \delta_{1},\delta_{2}\rangle }_{x}≜\delta_{1}^{⊤}M\left(x\right)\delta_{2}$ and the norm $\sqrt{{\langle \delta_{1},\delta_{2}\rangle }_{x}}$. A metric is called *uniformly bounded* if $a_{1}I\leq M\left(x\right)\leq a_{2}I$ holds ∀x and for some scalars $a_{2}\geq a_{1}>0$. Let Γ(a,b) be the set of smooth paths connecting two points *a* and *b* in $R^{n}$, where each c∈Γ(a,b) is a piecewise smooth mapping, $c:\left[0,1\right]\to R^{n}$, satisfying c(0)=a,c(1)=b. We use the notation c(s),s∈[0,1], and $c_{s}\left(s\right)≜\frac{\partial c}{\partial s}$. Given a metric M(x) and a curve c(s), we define the Riemannian energy of c(s) as $E\left(c\right)≜\int_{0}^{1}c_{s}^{⊤}M\left(c\left(s\right)\right)c_{s}\left(s\right)ds$. The Riemannian energy between *a* and *b* is defined as $E\left(a,b\right)≜\inf_{c\in \Gamma (a,b)}E\left(c\right)$.

Contraction theory [2] draws conclusions on the convergence between pairs of state trajectories toward each other by studying the evolution of the distance between any two infinitesimally close neighbouring trajectories. CCM generalizes contraction analysis to the controlled dynamics setting in which the analysis jointly searches for a controller and a metric that describes the contraction properties of the resulting closed-loop system. Following [1,14], we now briefly review CCMs by considering the nominal, i.e., uncertainty-free, system:

(7) $$\dot{x}=f\left(x\right)+B\left(x\right)u,$$

where $x\left(t\right)\in R^{n}$ and $u\left(t\right)\in R^{m}$. The differential form of (7) is given by ${\dot{\delta }}_{x}=A\left(x,u\right)\delta_{x}+B\left(x\right)\delta_{u},$ where $A\left(x,u\right)≜\frac{\partial f}{\partial x}+\sum_{i=1}^{m}\frac{\partial b_{i}}{\partial x}u_{i}$ with $b_{i}\left(x\right)$ denoting the *i*th column of B(x). Consider a function $V\left(x,\delta_{x}\right)=\delta_{x}^{⊤}M\left(x\right)\delta_{x}$ for some positive definite metric M(x), which can be viewed as the Riemannian squared differential length at point *x*. Differentiating and imposing that the squared length decreases exponentially with rate 2λ, one obtains

(8) $$\dot{V}\left(x,\delta_{x}\right)=\delta_{x}^{⊤}\left(\;\langle MA\rangle +\dot{M}\right)\delta_{x}+2\delta_{x}^{⊤}MB\delta_{u}\leq -2\lambda \delta_{x}^{⊤}M\delta_{x},$$

where $\dot{M}=\partial_{f+Bu}M=\partial_{f}M+\sum_{i=1}^{m}\partial_{b_{i}}Mu_{i}$. We first recall some basic results related to CCM.

**Definition** **1** ([1]). *The system (7) is said to be universally exponentially stabilizable if, for any feasible desired trajectory $x^{⋆}\left(t\right)$ and $u^{⋆}\left(t\right)$, a feedback controller can be constructed that for any initial condition x(0), a unique solution to (7) exists and satisfies $\|x\left(t\right)-x^{⋆}\left(t\right)\|\leq R\|x\left(0\right)-x^{⋆}\left(0\right)\|e^{-\lambda t},$ where λ and R are the convergence rate and overshoot, respectively, independent of the initial conditions.*

**Lemma** **1** ([1]). *If there exists a uniformly bounded metric M(x), i.e., $\alpha_{1}I\;\leq \;M\left(x\right)\;\leq \;\alpha_{2}I$ for some positive constants $\alpha_{1}$ and $\alpha_{2}$, such that for all x and $\delta_{x}\;\neq \;0$ satisfying $\delta_{x}^{⊤}MB=0$,*

(9a) $$\begin{array}{cc} \; & \delta_{x}^{⊤}\;\left(\;\langle M\frac{\partial f}{\partial x}\;\rangle \;+\;\partial_{f}M\;+\;2\lambda M\;\right)\;\delta_{x}\leq 0, \end{array}$$

(9b) $$\begin{array}{cc} \; & \delta_{x}^{⊤}\;\left(\;\langle M\frac{\partial b_{i}}{\partial x}\;\rangle \;+\;\partial_{b_{i}}M\right)\delta_{x}=0,\;i=1,\cdots ,m \end{array}$$

*hold, then the system (7) is universally exponentially stabilizable in the sense of Definition 1 via continuous feedback defined almost everywhere, and everywhere in the neighborhood of the target trajectory with the convergence rate λ and overshoot $R=\sqrt{\frac{\alpha_{2}}{\alpha_{1}}}$.*

The condition (9) ensures that the dynamics orthogonal to the input are contracting, i.e., (8) holds in the presence of $\delta_{x}^{⊤}MB=0$ and is often termed as the strong CCM condition [1]. In particular, the condition (9b) can be satisfied by enforcing that each column of B(x) forms a killing vector field for the metric M(x), i.e., $\langle \;M\frac{\partial b_{i}}{\partial x}\;\rangle \;+\;\partial_{b_{i}}M=0$ for all i=1,⋯,m. The CCM condition (9) can be transformed into a convex constructive condition for the metric M(x) by a change of variables. Let $W\left(x\right)=M^{-1}\left(x\right)$ (commonly referred to as the *dual metric*), and $B_{⊥}\left(x\right)$ be a matrix whose columns span the null space of the input matrix *B* (i.e., $B_{⊥}^{⊤}B=0$). Then, condition (9) can be cast as convex constructive conditions for W(x):

(10a) $$\begin{array}{cc} \; & B_{⊥}^{⊤}\;\left(\;\langle \;\frac{\partial f}{\partial x}W\;\rangle -\partial_{f}W+2\lambda W\;\right)\;B_{⊥}\leq 0 \end{array}$$

(10b) $$\begin{array}{cc} \; & \langle \;\frac{\partial b_{i}}{\partial x}W\;\rangle \;-\;\partial_{b_{i}}W=0,\;\mathrm{for}\;i=1,\cdots ,m. \end{array}$$

The existence of a contraction metric M(x) is sufficient for stabilizability via Lemma 1. What remains is constructing a feedback controller that achieves the universal exponential stabilizability (UES). As mentioned in [1,16], one way to derive the controller is to interprete the Riemann energy, $E(x^{⋆}\left(t\right),x\left(t\right))$, as an incremental control Lyapunov function and use it to construct a min-norm controller that renders for any time *t*

(11) $$\dot{E}\left(x^{⋆}\left(t\right),x\left(t\right)\right)\leq -2\lambda E\left(x^{⋆}\left(t\right),x\left(t\right)\right).$$

Specifically, at any time t>0, given the metric M(x) and a desired/actual state pair $\left(x^{⋆}\left(t\right),x\left(t\right)\right)$, a minimum-energy path, i.e., a geodesic, γ(·,t) connecting these two states (i.e., $\gamma \left(0,t\right)=x^{⋆}\left(t\right)$ and γ(1,t)=x(t)), can be computed (e.g., using the pseudospectral method in [21] to solve a nonlinear programming problem). Consequently, the Riemannian energy of the geodesic is defined as $E\left(x^{⋆}\left(t\right),x\left(t\right)\right)=\int_{0}^{1}\gamma_{s}{\left(s,t\right)}^{⊤}M\left(\gamma \left(s,t\right)\right))\gamma_{s}\left(s,t\right)ds$, where $\gamma_{s}\left(s\right)≜\frac{\partial \gamma }{\partial s}$, can be calculated. As noted in [16], from the formula for the first variation of energy [22], $\dot{E}\left(x^{⋆}\left(t\right),x\left(t\right)\right)=2\gamma_{s}^{⊤}\left(1,t\right)M\left(x\left(t\right)\right)\dot{x}\left(t\right)-2\gamma_{s}^{⊤}\left(0,t\right)M\left(x^{⋆}\left(t\right)\right){\dot{x}}^{⋆}\left(t\right)$. Therefore, (11) can be rewritten as

(12) $$\gamma_{s}^{⊤}\left(1,t\right)M\left(x\left(t\right)\right)\dot{x}\left(t\right)-\gamma_{s}^{⊤}\left(0,t\right)M\left(x^{⋆}\left(t\right)\right){\dot{x}}^{⋆}\left(t\right)\leq -\lambda E\left(x^{⋆}\left(t\right),x\left(t\right)\right),$$

where $\dot{x}\left(t\right)=f\left(x\left(t\right)\right)+B\left(x\left(t\right)\right)u\left(t\right)$ and ${\dot{x}}^{⋆}\left(t\right)=f\left(x^{⋆}\left(t\right)\right)+B\left(x^{⋆}\left(t\right)\right)u^{⋆}\left(t\right)$. Therefore, the control signal with a minimum norm for $u\left(t\right)-u^{⋆}\left(t\right)$ can then be obtained by solving the following quadratic programming (QP) problem:

(13) $$u\left(t\right)=\underset{k\in R^{m}}{\mathrm{argmin}}\|{k-u^{⋆}\left(t)\right\|}^{2}\mathrm{subject}\;\mathrm{to}\;(12)$$

at each time *t*, which is guaranteed to be feasible under condition (9) [1]. The minimization problem (13) is often termed as the *pointwise min-norm control* problem and has an analytic solution [23]. The above discussions can be summarized in the following theorem. The proof is trivial by following Lemma 1 and the subsequent discussions and is thus omitted.

**Theorem** **1** ([1]). *Given a nominal system (7), assume that there exists a uniformly bounded metric W(x) that satisfies (10) for all $x\in R^{n}$. Then, the control law constructed by solving (13) with $M\left(x\right)=W^{-1}\left(x\right)$, universally exponentially stabilizes the system (7) in the sense of Definition 1, where $R=\sqrt{\frac{\alpha_{2}}{\alpha_{1}}}$ with $\alpha_{1}$ and $\alpha_{2}$ being two positive constants satisfying $\alpha_{1}I\;\leq \;M\left(x\right)\;\leq \;\alpha_{2}I$.*

**Remark** **2.** *According to Definition 1 and Theorem 1, under the conditions of Theorem 1, given any feasible trajectory ($x^{⋆}(\left(\cdot \right),u^{⋆}\left(\cdot \right)$) of (7), a controller can always be constructed to ensure that the actual state trajectory x(·) exponentially converges to $x^{⋆}\left(\cdot \right)$.*

## 3. Robust Trajectory Tracking Using CCM and Disturbance Estimation

**Figure 1:**
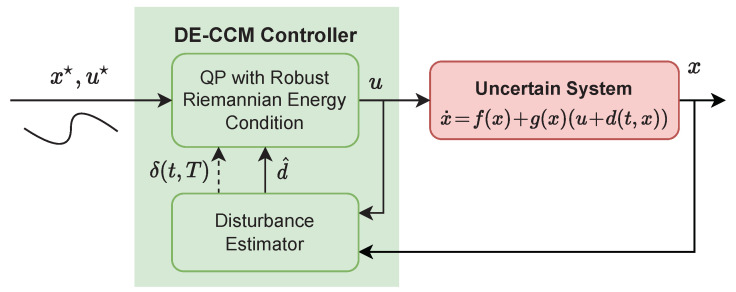
Block diagram of the closed-loop system with the proposed DE-CCM controller.

In Section 2, we have shown that existence of a CCM for a *nominal* (i.e., uncertainty-free) system can be used to construct a feedback control law to guarantee the universal exponential stabilizability (UES) of the system. In this section, we present a controller based on CCM and disturbance estimation to ensure the UES of the uncertain system (1), whose architecture is depicted in Figure 1.

### 3.1. CCMs for the Actual System

To apply the contraction method to design a controller to guarantee the UES of the uncertain system (1), we need to first search a valid CCM for it. Following Section 2, we can derive the counterparts of the strong CCM condition (9) or (10). Due to the particular structure with (1) attributed to the matched uncertainty assumption, we have the following lemma. A similar observation has been made in [14] for the case of matched parametric uncertainties. The proof is straightforward and thus omitted. One can refer to [14] for more details.

**Lemma** **2.** *The strong (dual) CCM condition for the uncertain system (1) is the same as the strong (dual) CCM condition, i.e., (9) and (10), for the nominal system.*

**Remark** **3.** *As a result of Lemma 2, a metric M(x) (dual metric W(x)) satisfying the condition (9) and (10) for the nominal system (7) is always a CCM (dual CCM) for the true system (1).*

Define $D=\{y\in R^{m}:\|y\|\leq b_{d}\}$, where $b_{d}$ is introduced in Assumption 1. Assumption 1 indicates d(t,x)∈D for any t≥0 and x∈X. As mentioned in Section 2, given a CCM and a desired trajectory $x^{⋆}\left(t\right)$ and $u^{⋆}\left(t\right)$ for a nominal system, a control law can be constructed to ensure exponential convergence of the actual state trajectory x(t) to the desired state trajectory $x^{⋆}\left(t\right)$. In practice, we have access to only the nominal dynamics (5) instead of the true dynamics to plan a trajectory $x^{⋆}\left(t\right)$ and $u^{⋆}\left(t\right)$. The following lemma gives the condition when $x^{⋆}\left(t\right)$, planned using the nominal dynamics (5), is also a feasible state trajectory for the true system.

**Lemma** **3.** *Given a desired trajectory $x^{⋆}\left(t\right)$ and $u^{⋆}\left(t\right)$ satisfying the nominal dynamics (5) with $x^{⋆}\left(t\right)\in X$, if *

(14) $$u^{⋆}\left(t\right)\in U⊖D,\;\forall t\geq 0,$$

*then $x^{⋆}\left(t\right)$ is also a feasible state trajectory for the true system (1).*

**Proof**. Define ${\bar{u}}^{⋆}\left(t\right)≜u^{⋆}\left(t\right)-d\left(t,x^{⋆}\left(t\right)\right)$. Since $u^{⋆}\left(t\right)\in U⊖D$ and $-d(t,x^{⋆}\left(t\right))\in D$, which is due to $x^{⋆}\left(t\right)\in X$ and Assumption 1, we have ${\bar{u}}^{⋆}\left(t\right)\in U$. By comparing the dynamics in (1) and (5), we conclude that $x^{⋆}\left(t\right)$ and ${\bar{u}}^{⋆}\left(t\right)$ satisfy the true dynamics (1) and thus are a feasible state and input trajectory for the true system. □

Lemma 3 provides a way to verify whether a trajectory planned using the nominal dynamics is a feasible trajectory for the true system in the presence of actuator limits. In the absence of such limits, any feasible trajectory for the learned dynamics is also a feasible trajectory for the true dynamics due to the particular structure of (1) associated with the matched uncertainty assumption.

### 3.2. Robust Riemannian Energy Condition

Section 2 shows that, given a nominal system and a CCM for such a system, a control law can be constructed via solving a QP problem (13) with a condition to constrain the decreasing rate of the Riemannian energy, i.e., condition (12). When considering the uncertain dynamics in (1), the condition (12) becomes

(15) $$\gamma_{s}^{⊤}\left(1,t\right)M\left(x\left(t\right)\right)\dot{x}\left(t\right)-\gamma_{s}^{⊤}\left(0,t\right)M\left(x^{⋆}\left(t\right)\right){\dot{x}}^{⋆}\left(t\right)\leq -\lambda E\left(x^{⋆}\left(t\right),x\left(t\right)\right),$$

where $\dot{x}\left(t\right)=f\left(x\left(t\right)\right)+B\left(x\left(t\right)\right)\left(u\left(t\right)+d\left(x\left(t\right)\right)\right)$ represents the true dynamics evaluated at x(t), and ${\dot{x}}^{⋆}\left(t\right)=f\left(x^{⋆}\right)+B\left(x^{⋆}\right)u^{⋆}$ as defined in (5). Several observations follow immediately. First, it is clear that (15) is *not implementable* due to its dependence on the true uncertainty d(x(t)) through $\dot{x}\left(t\right)$. Second, if we could have access to the *pointwise value* of d(x(t)) at each time *t*, (15) will become implementable even when we do not know the exact functional representation of d(x). Third, if we could estimate the pointwise value of d(x(t)) at each time *t* with a bound to quantify the estimation error, then we could derive a robust condition for (15). Specifically, assume d(x(t)) is estimated as $\hat{d}\left(t\right)$ at each time *t* with a uniform estimation error bound (EEB) δ, i.e., $\|\hat{d}\left(t\right)-d\left(x\left(t\right)\right)\|\leq \delta ,\;\forall t\geq 0.$ Then, we could immediately get the following sufficient condition for (15):

(16) $$\begin{array}{cc} \gamma_{s}^{⊤}\left(1,t\right) & M\left(x\right)\dot{\overset{ˇ}{x}}\left(t\right)-\gamma_{s}^{⊤}\left(0,t\right)M\left(x^{⋆}\right){\dot{x}}^{⋆}+\|\gamma_{s}^{⊤}\;\left(1,t\right)M\left(x\right)B\left(x)\right\|\delta \leq -\lambda E\left(x^{⋆},x\right), \end{array}$$

where

(17) $$\dot{\overset{ˇ}{x}}\left(t\right)≜f\left(x\right)+B\left(x\right)\left(u\left(t\right)+\hat{d}\left(t\right)\right).$$

Moreover, since M(x) satisfies the CCM condition (9), u(t) that satisfies (16) is guaranteed to exist for any t≥0, regardless of the size of δ, if the input constraint set U is sufficiently large. We term condition (16) the *robust Riemannian energy* (RRE) condition.

### 3.3. Disturbance Estimation with a Computable EEB

We now introduce a disturbance estimation scheme to estimate the pointwise value of the uncertainty d(x) with a pre-computable EEB, which can be systematically improved by tuning a parameter in the estimation law. The estimation scheme is based on the piecewise-constant estimation (PWCE) law in [24], which was originally from [25]. The PWCE law consists of two elements, namely a state predictor and a piecewise-constant update law. The state predictor is defined as:

(18) $$\dot{\hat{x}}\left(t\right)=f\left(x\left(t\right)\right)+B\left(x\left(t\right)\right)u\left(t\right)+\hat{\sigma }\left(t\right)-a\tilde{x}\left(t\right),\;\hat{x}\left(0\right)=x\left(0\right),$$

where $\tilde{x}\left(t\right)≜\hat{x}\left(t\right)-x\left(t\right)$ is the prediction error, and *a* is an arbitrary positive constant. The estimation, $\hat{\sigma }\left(t\right)$, is updated in a piecewise-constant way:

(19) $$\left\{\begin{array}{cc} \hat{\sigma }\left(t\right)\; & =\hat{\sigma }\left(iT\right),\;t\in \left[iT,\left(i+1\right)T\right),\\ \hat{\sigma }\left(iT\right)\; & =-\frac{a}{e^{aT}-1}\tilde{x}\left(iT\right), \end{array}\right.$$

where *T* is the estimation sampling time, and i=0,1,2,⋯. Finally, the pointwise value of d(x(t)) at time *t* is estimated as

(20) $$\hat{d}\left(t\right)=B^{†}\left(x\left(t\right)\right)\hat{\sigma }\left(t\right),$$

where $B^{†}\left(x\left(t\right)\right)$ is the pseudoinverse of B(x(t)). The following lemma establishes the EEB associated with the estimation scheme in (18) and (19). The proof is similar to that in [24]. For completeness, it is given in Appendix A.

**Lemma** **4.** *Given the dynamics (1) subject to Assumption 1, and the estimation law in (18) and (19), if x∈X and u∈U for any t≥0, the estimation error can be bounded as   *

(21) $$\begin{array}{cc} \left\|\hat{d}\left(t\right)\;-\;d\left(t,x\left(t\right)\right)\right\| & \leq \delta \left(t,T\right)≜\left\{\begin{array}{c} \;\;\;b_{d},\;\forall \;0\leq t<T,\\ \;\;\;\alpha \left(T\right)\max_{x\in X}B^{†}\left(x\right),\;\forall \;t\geq T, \end{array}\right. \end{array}$$

*where*

(22) $$\begin{array}{cc} \alpha (T) & ≜\left(2\sqrt{n}T\left(L_{d}\phi +l_{d}\right)+\left(1\;-\;e^{-aT}\right)\sqrt{n}b_{d}\right)\max_{x\in X}\|B(x)\|+2\sqrt{n}TL_{B}b_{d}, \end{array}$$

(23) $$\begin{array}{cc} \phi & ≜\max_{x\in X,u\in U}\|f(x)+B(x)u\|+b_{d}\max_{x\in X}\left\|B(x)\right\|, \end{array}$$

*with constants $L_{B}$, $L_{d}$ and $b_{d}$ from Assumption 1, and ϕ defined in (23). Moreover, $\lim_{T\to 0}\delta \left(t,T\right)=0,$ for any t≥T.*

**Proof**. See Appendix A. □

**Remark** **4.** *Lemma 4 implies that theoretically, for t≥T, the disturbance estimation after a single sampling interval can be made arbitrarily accurate by reducing T, which further indicates that the conservatism with the RRE condition can be arbitrarily reduced after a sampling interval.*

In practice, the value of *T* is subject to the limitations related to computational hardware and sensor noise. Additionally, using a very small *T* tends to introduce high frequency components in the control loop, potentially harming the robustness of the closed-loop system, e.g., against time delay. This is similar to the use of a high adaptation rate in model reference adaptive control schemes as discussed in [13]. Therefore, one should avoid the use of a very small *T* for the sake of robustness unless a low-pass filter is used to filter the estimated disturbance before fed into (16), as suggested by the $L_{1}$ adaptive control theory [13].

**Remark** **5.** *The estimation in [0,T) cannot be arbitrarily accurate. This is because the estimation in [0,T) depends on $\tilde{x}\left(0\right)$ according to (19). Considering that $\tilde{x}\left(0\right)$ is purely determined by the initial state of the system, x(0), and the initial state of the predictor, $\hat{x}\left(0\right)$, it does not contain any information of the uncertainty. Since T is usually very small in practice, lack of a tight estimation error bound for the interval [0,T) will not cause an issue from a practical point of view. Additionally, the estimation of ϕ defined in (23) could be quite conservative. Further considering the frequent use of Lipschitz continuity and inequalities related to matrix/vector norms in deriving the constant α(T), α(T) can be overly conservative. Therefore, for practical implementation, one should leverage some empirical study, e.g., performing simulations under a few user-selected functions of d(t,x) and determining a bound for δ(t,T). In our experiments, we found the theoretical bound δ(t,T) computed according to (21) was usually at least 10 and could be $10^{4}$ times more conservative.*

### 3.4. Exponentially Convergent Trajectory Tracking

Based on the review of contraction control in Section 2 and the discussions in Section 3.2 and Section 3.3, the control law can be obtained by solving the following QP problem at each time *t*:

(24) $$u\left(t\right)=\underset{k\in R^{m}}{\mathrm{argmin}}\|{k-u^{⋆}\left(t\right)\|}^{2}$$

subject to

(25) $$\gamma_{s}^{⊤}\left(1,t\right)M\left(x\right)\dot{\overset{ˇ}{x}}-\gamma_{s}^{⊤}\left(0,t\right)M\left(x^{⋆}\right){\dot{x}}^{⋆}+\|\gamma_{s}^{⊤}\;\left(1,t\right)M\left(x\right)B\left(x\right)\|\delta \left(t,T\right)\leq -\lambda E\left(x^{⋆},x\right),$$

where $\dot{\overset{ˇ}{x}}\left(t\right)=f\left(x\right)+B\left(x\right)\left(k+\hat{d}\left(t\right)\right)$, according to (17), depends on $\hat{d}\left(t\right)$, which is from the disturbance estimation law defined by (18) to (20), δ(t,T) as defined in (21), and ${\dot{x}}^{⋆}\left(t\right)=f\left(x^{⋆}\right)+B\left(x^{⋆}\right)u^{⋆}$ as defined in (5). Similar to (13),  problem (24) is a pointwise min-norm control problem and has an analytic solution [23]. Specifically, denoting $\phi_{0}\left(t,x^{⋆},x\right)≜\gamma_{s}^{⊤}\left(1,t\right)M\left(x\right)(f\left(x\right)+B\left(x\right)\left(u^{⋆}\left(t\right)+\hat{d}\left(t\right)\right)+\|\gamma_{s}^{⊤}\;\left(1,t\right)M\left(x\right)B\left(x\right)\|\delta \left(t,T\right)-\gamma_{s}^{⊤}\left(0,t\right)M\left(x^{⋆}\right){\dot{x}}^{⋆}+\lambda E\left(x^{⋆},x\right)$ and $\phi_{1}\left(x^{⋆},x\right)≜B^{⊤}\left(x\right)M\left(x\right)\gamma_{s}\left(1,t\right)$, (25) can be written as $\phi_{0}\left(t,x^{⋆},x\right)+\phi_{1}^{⊤}\left(x^{⋆},x\right)\left(k-u^{⋆}\left(t\right)\right)\leq 0$, and the solution for (24) is given by

(26) $$\;u\left(t\right)=k^{⋆}\;=\;\left\{\begin{array}{cc} u^{⋆}\left(t\right)\; & \;\mathrm{if}\;\phi_{0}\left(t,x^{⋆},x\right)\;\leq \;0,\\ u^{⋆}\left(t\right)-\;\frac{\phi_{0}\left(t,x^{⋆},x\right)\phi_{1}\left(x^{⋆},x\right)}{{\left\|\phi_{1}\left(x^{⋆},x\right)\right\|}^{2}} & \;\mathrm{if}\;\phi_{0}\left(t,x^{⋆},x\right)\;>\;0. \end{array}\right.$$

To move forward with analysis, we need to verify that when $x\left(t\right),x^{⋆}\left(t\right)\in X$, the control signal u(t) resulting from solving the QP problem (24) satisfies u(t)∈U. Deriving verifiable conditions to ensure this set bound is outside the scope of this paper and will be addressed as future work. We are now ready to state the main result of the paper.

**Theorem** **2.** *Given an uncertain system represented by (1) satisfying Assumption 1, assume that there exists a metric W(x) such that for all x∈X, (10) holds and $\alpha_{1}I\leq M\left(x\right)=W^{-1}\left(x\right)\leq \alpha_{2}I$ holds for positive constants $\alpha_{1}$ and $\alpha_{2}$. Furthermore, suppose that a nominal trajectory ($x^{⋆}\left(\cdot \right),\;u^{⋆}\left(\cdot \right)$) planned using the nominal dynamics (5) and the initial actual states x(0) satisfy (14) and*

(27) $$\Omega \left(t\right)\;≜\;\left\{y\;\in \;R^{n}\;:y\leq \|x^{⋆}\left(t\right)\|\;+\;\sqrt{\frac{\alpha_{2}}{\alpha_{1}}}\|x\left(0\right)\;-\;x^{⋆}\left(0\right)\|e^{-\lambda t}\right\}\subset X,$$

*for any t≥0. Then, if u(t) from solving (24) satisfies u(t)∈U for any t≥0, the control law constructed by solving (24) ensures x(t)∈X for any t≥0, and furthermore, universally exponentially stabilizes the uncertain system (1) in the sense of Definition 1 with $R=\sqrt{\frac{\alpha_{2}}{\alpha_{1}}}$, i.e.,*

(28) $$\|x\left(t\right)-x^{⋆}\left(t\right)\|\leq \sqrt{\frac{\alpha_{2}}{\alpha_{1}}}\|x\left(0\right)-x^{⋆}\left(0\right)\|e^{-\lambda t},\;\forall t\geq 0.$$

**Proof**. We use contradiction to show x(t)∈X for all t≥0. Assume this is not true. According to (27), x(0)∈X. Since x(t) is continuous, there must exist a time τ such that

(29) $$x\left(t\right)\in X,\;\forall t\in \left[0,\tau^{-}\right]\;\mathrm{and}\;x\left(\tau \right)\notin X.$$

Now let us consider the system evolution in $\left[0,\tau^{-}\right]$. Since u(t)∈U by assumption and x(t)∈X for any *t* in $\left[0,\tau^{-}\right]$, the EEB in (21) holds in $\left[0,\tau^{-}\right]$. As a result, the control law obtained from solving (24) ensures satisfaction of the RRE condition (16) and thus satisfaction of the Riemannian energy condition (15) for the uncertain system (1), and thereby universally exponentially stabilizes the uncertain system (1) in $\left[0,\tau^{-}\right]$, in the sense of Definition 1 with $R=\sqrt{\frac{\alpha_{2}}{\alpha_{1}}}$, according to Theorem 1. On the other hand, satisfaction of (14) implies that $x^{⋆}\left(t\right)$ is a feasible state trajectory for the uncertain system (1) according to Lemma 3. Further considering Theorem 1, we have $\|x(t)\|\leq \|x^{⋆}\left(t\right)\|+\sqrt{\frac{\alpha_{2}}{\alpha_{1}}}\|x\left(0\right)-x^{⋆}\left(0\right)\|e^{-\lambda t}$ for any *t* in $\left[0,\tau^{-}\right]$. Due to (27), the preceding inequality indicates that x(t) remains in the interior of X for *t* in $\left[0,\tau^{-}\right]$. This, together with the continuity of x(t), immediately implies x(τ)∈X, which contradicts (29). Therefore, we conclude that x(t)∈X for all t≥0. From the development of the proof, it is clear that with the control law given by the solution of (24), the UES of the closed-loop system in the sense of Definition 1 with $R=\sqrt{\frac{\alpha_{2}}{\alpha_{1}}}$ for all t≥0 is achieved, which is mathematically represented by (28). The proof is complete. □

### 3.5. Discussion

Theorem 2 essentially states that under certain assumptions, the proposed controller guarantees exponential convergence of the actual state trajectory x(t) to a desired one $x^{⋆}\left(t\right)$. With the exponential guarantee, if the actual trajectory meets the desired trajectory at certain time τ, then these two trajectories will stay together afterward. While the exponential convergence guarantee is stronger than the performance guarantees provided by existing adaptive CCM-based approaches [14,15] that deal with similar settings (i.e., matched uncertainties), the proposed method requires the knowledge of the Lipschitz bound of the uncertainty d(t,x) and the input matrix function B(x) to be in a compact set known a priori (see Assumption 1), and the actual control inputs to stay in a compact set known a priori, which cannot be verified at this moment due to the lack of a bound on the control inputs. These requirements are not needed in [14,15].

The approach here is related to the robust control Lyapunov-based approaches [23] which provide robust stabilization around an equilibrium point (as opposed to a trajectory considered in this paper) in the presence of uncertainties.

**Remark** **6.** *The exponential convergence guarantee stated in Theorem 2 is based on a continuous-time implementation of the controller. In practice, a controller is normally implemented on a digital processor or controller with a fixed sampling time. As a result, the property of exponential convergence may be slightly violated.*

Computational cost: As can be seen from Section 2 and Section 3.2, Section 3.3 and Section 3.4, computation of the control signal at each time *t* includes three steps: (i) updating the estimated disturbance $\hat{d}\left(t\right)$ via (18) to (20), (ii) computing the geodesic γ(·,t) connecting the actual and nominal states (see the discussion below (11)), and (iii) computing the control signal u(t) via (26). The computation costs of steps (i) and (iii) are quite low as they only involve integration and algebraic calculation. In comparison, step (ii) has a relatively high computational cost as it necessitates solving a nonlinear programming (NLP) problem. However, since the NLP problem does not involve dynamic constraints, it is much easier to solve than a nonlinear model predictive control (MPC) problem [21]. Following [21], such a problem can be efficiently solved by applying a pseudospectral method.

## 4. Simulation Results

In this section, we illustrate the performance of our proposed tracking controller based on the RRE condition and disturbance estimation, denoted as DE-CCM, using aircraft and planar quadrotor examples. For both examples, we perform comparisons of DE-CCM with standard CCM controllers that ignore the uncertainties and adaptive CCM (Ad-CCM) controllers considering parametric uncertainties designed using the approach in [14]. All the computations and simulations were performed in Matlab R2021b.

### 4.1. Longitudinal Dynamics of an Aircraft

We first implement our method on the simplified pitch dynamics of an aircraft borrowed from [26]:

(30) $$\dot{x}≜\left[\begin{array}{c} \dot{\theta }\\ \dot{\alpha }\\ \dot{q} \end{array}\right]=\left[\begin{array}{c} q\\ q-\bar{L}\left(\alpha \right)\\ -k_{q}q+\bar{M}\left(\alpha \right) \end{array}\right]+\left[\begin{array}{c} 0\\ 0\\ 1 \end{array}\right]u,$$

where θ, α and *q* are the pitch angle (in rad), angle of attack (in rad), and pitch rate (in rad/s). Here $\bar{L}\left(\alpha \right)$ and $\bar{M}\left(\alpha \right)$ are aerodynamic lift and moment, respectively. Using the flat plat theory, these two aerodynamic terms are approximated by $\bar{L}\left(\alpha \right)=0.8\sin\left(2\alpha \right)$ and $\bar{M}\left(\alpha \right)=-l_{\alpha }\bar{L}\left(\alpha \right)$ with unknown parameters $k_{q}\in \left[0.1\;0.8\right]$ and $l_{\alpha }\in \left[-3\;1\right]$. For all the simulations, the true values are chosen to be $k_{q}=0.8$, $l_{\alpha }=-3$, while the nominal values of these parameters used in designing all the tested controllers are limited to $k_{q}^{nom}=0.2$, $l_{\alpha }^{nom}=-1$. As a result, the dynamics can be recast in the form of (30) with $f\left(x\right)={\left[q,q-\bar{L}\left(\alpha \right),-k_{q}^{nom}q-l_{\alpha }^{nom}\bar{L}\left(\alpha \right)\right]}^{⊤}$, $B\left(x\right)={\left[0,0,1\right]}^{⊤}$ and $d\left(t,x\right)=-\left(k_{q}-k_{q}^{nom}\right)q-\left(l_{\alpha }-l_{\alpha }^{nom}\right)\bar{L}\left(\alpha \right)$. The control objective is to drive the system from nominal initial states ${\left[0,0,0\right]}^{T}$ to terminal states ${\left[180,0,0\right]}^{T}$. For CCM search and trajectory planning, the following constraints are enforced: x∈X=[−10°,180°]×[−5°,40°]×[−10,50]°/s, u∈U=[−15,15]°/s${}^{2}$.

We set the convergence rate λ to 1. By gridding the set of α and evaluating the constraints (9) in those grid points, we found a CCM W(x) as a quadratic function of α with the SPOT toolbox [27] (to formulate the convex optimization problem) and Mosek solver [28]. Additionally, the constants $\alpha_{1}$ and $\alpha_{2}$ in (28) such that $\alpha_{1}I\leq M\left(x\right)=W^{-1}\left(x\right)\leq \alpha_{2}I$ for all x∈X were found to be $\alpha_{1}=0.1$ and $\alpha_{2}=396.5$. We planned a nominal trajectory ($x^{⋆}\left(\cdot \right)$, $u^{⋆}\left(\cdot \right)$) using OptimTraj [29,30], to drive the system from the initial states ${\left[0,0,0\right]}^{T}$ to the terminal states ${\left[180{}^{\circ},0,0\right]}^{T}$, while minimizing the task completion time ($T_{a}$) and energy consumption characterized by the cost function $J=\int_{0}^{T_{a}}{u(t)}^{2}dt+5T_{a}$. For simulation, OPTI [31] and Matlab fmincon solvers were used to solve the geodesic optimization problem (see Section 2). The initial states of the actual system were chosen to be ${\left[5{}^{\circ},5{}^{\circ},0\right]}^{T}$, slightly deviated from that planned ones to better illustrate the tracking performance. We implemented our proposed DE-CCM, Ad-CCM from [14] and a standard CCM which neglects all the uncertainty. For Ad-CCM design, the adaptation gain was chosen to be diag $\left(\left[10^{3},10^{3}\right]\right)$ to achieve a relatively good tracking result, while further increasing it did not help much with the tracking performance. The design procedure for Ad-CCM in [14] requires a parametric structure for the uncertainty, which is given by

(31) $$d\left(t,x\right)=\underset{\Delta (t,x)}{\underset{︸}{\left[\begin{array}{cc} q & \bar{L}\left(\alpha \right) \end{array}\right]}}\underset{\theta }{\underset{︸}{\left[\begin{array}{c} k_{q}^{nom}-k_{q}\\ l_{\alpha }^{nom}-l_{\alpha } \end{array}\right]}}=\left[\begin{array}{cc} q & 0.8\sin(2\alpha ) \end{array}\right]\left[\begin{array}{c} -0.6\\ 2 \end{array}\right],$$

where ∆(t,x) is the known base function and θ is the unknown parameter vector to be estimated by the adaptive law proposed in [14]. The control signals under all three controllers were updated at 200 Hz.

It is easy to notice from Assumption 1 that $L_{B}=0$ and $l_{d}=0$ since the input matrix is constant, and the uncertainty is time-invariant. We can also verify that the disturbance is bounded by $b_{d}=2.12$ and has a Lipschitz constant $L_{d}=3.80$. By gridding the space X and making use of the control input bound, the system derivative can also be bounded by a constant ϕ=2.11. According to (21), if we want to achieve an EEB δ(t,T)=0.05 for all t≥T, the maximum value for the estimation sample time *T* is $7.76\times 10^{-4}$ s. However, as mentioned in Remark 5, the way to compute the EEB is quite conservative. In the simulations we found that $T_{s}=0.005$ was more than enough to ensure the EEB and therefore used $T_{s}=0.005$ for implementing the DE-CCM controller.

**Figure 2:**
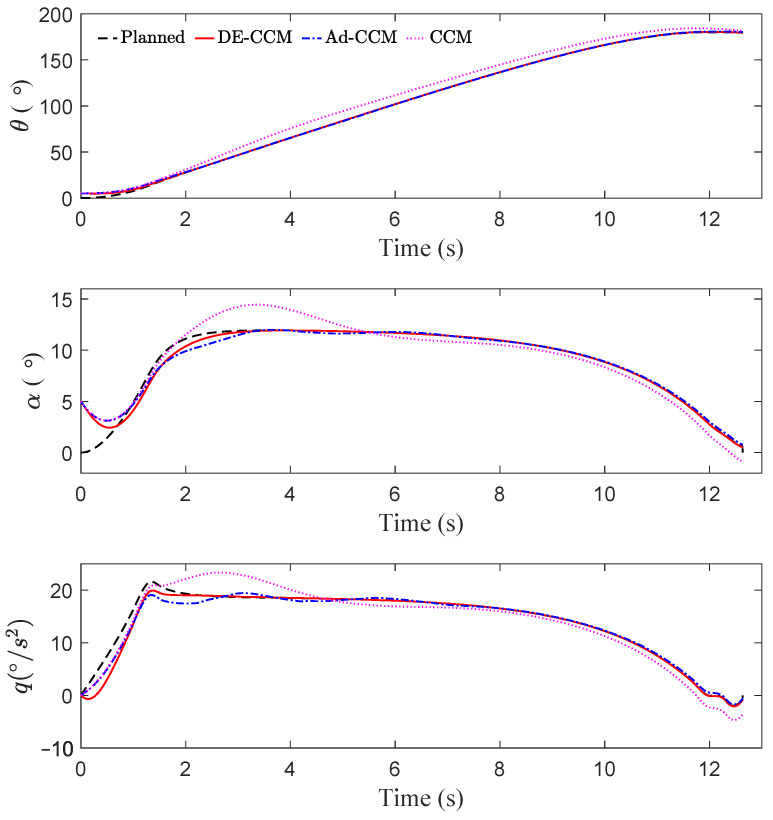
Trajectory tracking performance of different controllers.

**Figure 3:**
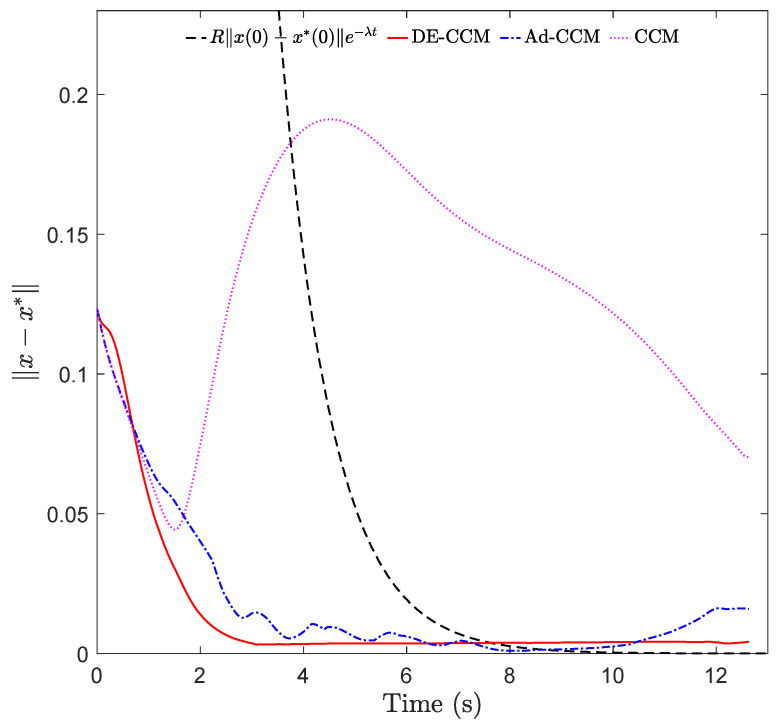
Tracking error under different controllers.

As shown in Figure 2 and Figure 3, due to ignoring the uncertainties, CCM yielded a large tracking error between 2 and 6 s. The state trajectories under Ad-CCM had some oscillations, which lasted roughly up to 8 s. All three states yielded by DE-CCM achieve good tracking performance without large deviations from the planned trajectories, unlike the performance yielded by Ad-CCM and CCM. From Figure 3 we notice that the tracking error represented by $x-x^{⋆}$ under DE-CCM monotonically decreases and achieves the smallest steady-state error. The small non-zero tracking error at the end under DE-CCM, which is inconsistent with the performance guarantee in (25), is due to the limited control update frequency, while the performance guarantee in Lemma 4 holds under continuous update of the control signal, i.e., corresponding to an infinitely high update frequency. Table 1 shows the mean squared error (MSE) for state trajectory tracking defined by

(32) $$\mathrm{MSE}=\frac{1}{N}\sum_{i=1}^{N}\|{x\left(t_{i}\right)-x^{⋆}\left(t_{i}\right)\|}^{2},$$

where *N* is the number of data points, under DE-CCM, Ad-CCM and CCM. We observe that DE-CCM outperforms CCM and Ad-CCM in terms of MSE by 54% and 2%, respectively.

**Figure 4:**
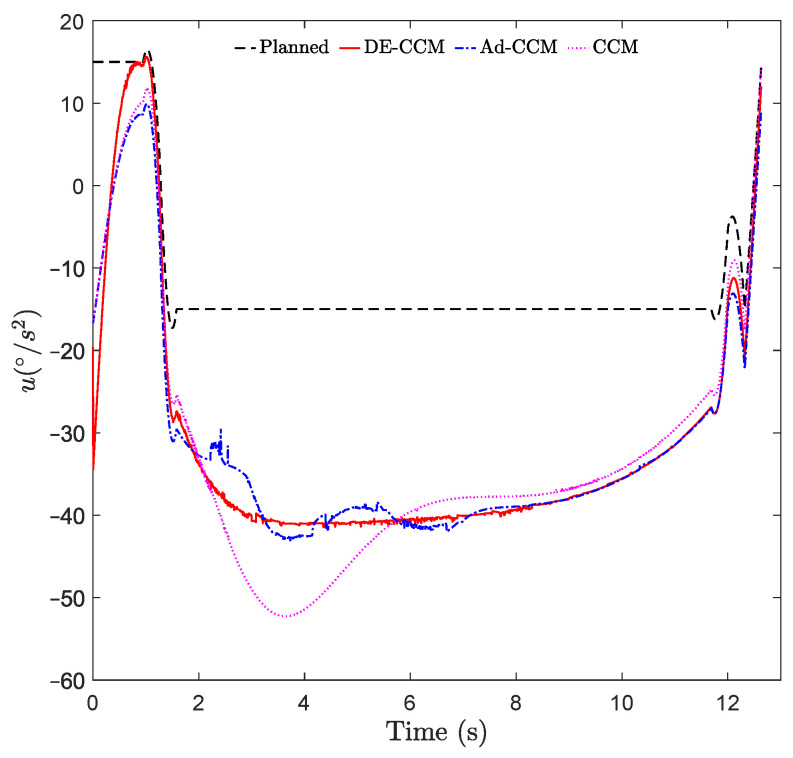
Control inputs yielded by different controllers.

From Figure 4, we observe that the input of DE-CCM is smoother than Ad-CCM. The small oscillations between 2 s to 8 s in DE-CCM input are due to the finite tolerance in the geodesic optimization. Decreasing the tolerance and the sample time will reduce the oscillations but request more iterations (and thus more time) to compute the control signal at each time step.

### 4.2. Planar Quadrotor

A planar quadrotor system is borrowed from [16]. The state vector is defined as $x={\left[p_{x},p_{z},\phi ,v_{x},v_{z},\dot{\phi }\right]}^{⊤}$, where $p_{x}$ and $p_{z}$ are the positions in *x* and *z* directions, respectively, $v_{x}$ and $v_{z}$ are the slip velocity (lateral) and the velocity along the thrust axis in the body frame of the vehicle, ϕ is the angle between the *x* direction of the body frame and the *x* direction of the inertia frame. The input vector $u=[u_{1},u_{2}]$ contains the thrust force produced by each of the two propellers. The dynamics of the vehicle are given by

$$\dot{x}=\left[\begin{array}{c} \dot{\;}p_{x}\\ \dot{\;}p_{z}\\ \dot{\phi }\\ \dot{\;}v_{x}\\ \dot{\;}v_{z}\\ \ddot{\phi } \end{array}\right]=\left[\begin{array}{c} v_{x}\cos\left(\phi \right)-v_{z}\sin\left(\phi \right)\\ v_{x}\sin\left(\phi \right)+v_{z}\cos\left(\phi \right)\\ \dot{\phi }\\ v_{z}\dot{\phi }-g\sin\left(\phi \right)\\ -v_{x}\dot{\phi }-g\cos\left(\phi \right)\\ 0 \end{array}\right]+\left[\begin{array}{cc} 0 & 0\\ 0 & 0\\ 0 & 0\\ 0 & 0\\ \frac{1}{m} & \frac{1}{m}\\ \frac{l}{J} & -\frac{l}{J} \end{array}\right]\left(u+d\left(t,x\right)\right),$$

where *m* and *J* denote the mass and moment of inertia about the out-of-plane axis, and *l* is the distance between each of the propellers and the vehicle center, and d(t,x) denotes the unknown disturbances exerted on the propellers. The parameters were set as m=0.486 kg, $J=0.00383\;Kgm^{2}$, and l=0.25 m. The uncertainty d(t,x) was set to be $d\left(t,x\right)=0.15\left(v_{x}^{2}+v_{z}^{2}\right)\left[-1+0.3\sin\left(2t\right);-1+0.3\cos\left(2t\right)\right]$. We imposed the following constraints: $x\in X≜\left[0,10\right]\times \left[0,10\right]\times \left[-\frac{\pi }{3},\frac{\pi }{3}\right]\times \left[-2,2\right]\times \left[-1,-1\right]\times \left[-\frac{\pi }{3},\frac{\pi }{3}\right]$, $u\in U≜\left[0,\frac{3}{2}mg\right]\times \left[0,\frac{3}{2}mg\right]$.

When searching for CCM, we parameterized the CCM *W* by ϕ and $v_{x}$ and imposed the constraint W≥0.01I. The convergence rate λ was chosen to be 0.8. More details about synthesizing the CCM can be found in [17]. For estimating the disturbance using (18) to (20), we set a=10. It is easy to verify that $L_{d}=1.23$, $l_{d}=0.64$, $b_{d}=1.38$, and $L_{B}=0$ (due to the fact that *B* is constant) satisfy (2). By gridding the space X, the constant ϕ in (23) can be determined as ϕ=584.6. According to (21), if we want to achieve an EEB δ(t,T)=0.1 for all t≥T, then the estimation sampling time needs to satisfy $T\leq 6.42\times 10^{-7}$ s. However, as noted in Remark 5, the EEB computed according to (21) could be overly conservative. In the simulations, we found that the estimation sampling time of 0.002 s was more than enough to ensure the desired EEB and therefore simply set T=0.002 s.

**Figure 5:**
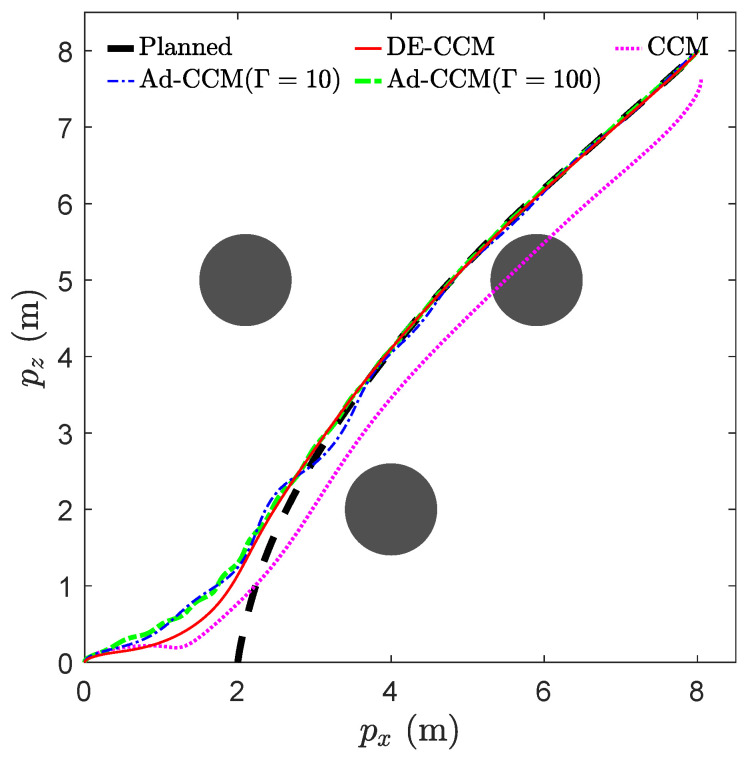
Trajectory tracking performance of different controllers.

We consider the task of navigation from (2,0) to (8,8) while avoiding three obstacles depicted by black circles in Figure 5. A nominal trajectory ($x^{⋆}\left(\cdot \right)$, $u^{⋆}\left(\cdot \right)$) was generated using OptimTraj [29] to minimize the cost $J=\int_{0}^{T_{a}}{u(t)}^{2}dt+5T_{a}$, where $T_{a}$ is the arrival time. OPTI [31] and Matlab fmincon solvers were used to solve the geodesic optimization problem (see Section 2). The actual start point was set to be (0,0), which was different from the planned start point, to reveal the trajectory convergence pattern.

**Figure 6:**
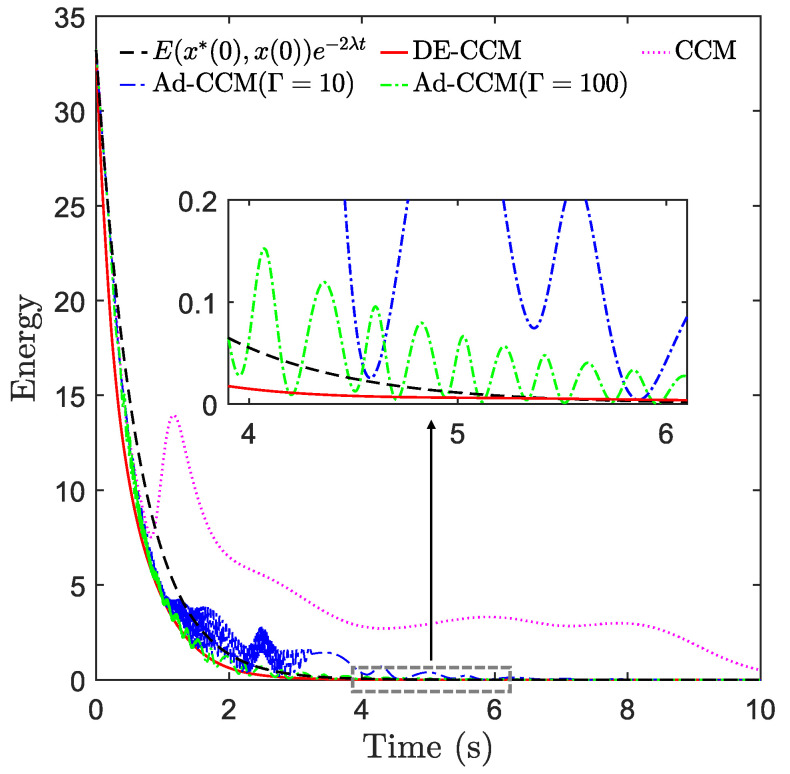
Riemannian energy under different controllers. $E_{0}≜E\left(x^{⋆}\left(0\right),x\left(0\right)\right)$.

**Figure 7:**
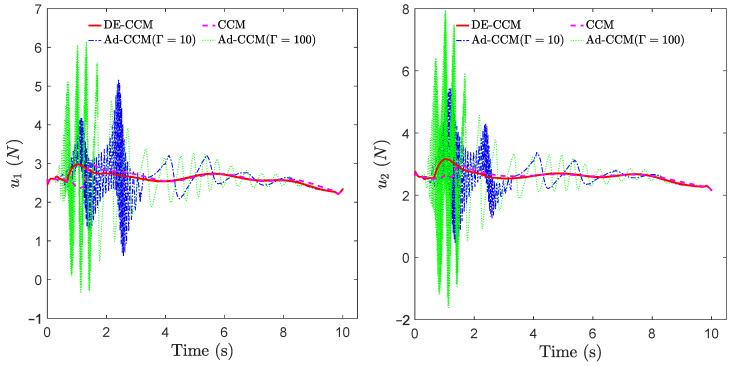
Control inputs yielded by different controllers.

For comparison, we also designed a standard CCM controller by completely ignoring the uncertainty and two adaptive CCM (Ad-CCM) controllers following the approach in [14]. To apply the approach in [14] which can only handle parametric uncertainties, we parameterized the uncertainty as

(33) $$d\left(t,x\right)=\underset{\Delta (t,x)}{\underset{︸}{\left[\begin{array}{cc} v_{x}^{2}\left(-1+0.3\sin\left(2t\right)\right) & v_{z}^{2}\left(-1+0.3\sin\left(2t\right)\right)\\ v_{x}^{2}\left(-1+0.3\cos\left(2t\right)\right) & v_{z}^{2}\left(-1+0.3\cos\left(2t\right)\right) \end{array}\right]}}\underset{\theta }{\underset{︸}{\left[\begin{array}{c} 0.15\\ 0.15 \end{array}\right]}},$$

where Δ(t,x) is the basis function that is assumed to be known, and θ is th vector of unknown parameters. With the parametric structure (33), we designed two adaptive CCM controllers using Γ=10 and Γ=100, respectively, where Γ denotes the adaptive gain. Figure 5 shows the planned and actual trajectories under the CCM, Ad-CCM, and our proposed controller based on the RRE condition and disturbance estimation, denoted as DE-CCM, while Figure 6 and Figure 7 show the control inputs and Riemannian energy. One can see that the actual trajectories yielded by the CCM controller deviated quite a lot from the planned ones and collided with one obstacle. On the other hand, the actual trajectories yielded by the DE-CCM controller converged to the desired trajectory as expected and almost overlapped with it afterward. In fact, the slight deviations of actual trajectories from the desired ones under the DE-CCM controller were due to the finite step size associated with the ODE solver used for the simulations (see Remark 6). Table 2 shows the MSE for state trajectory tracking defined in (32). We observe that DE-CCM outperforms CCM and Ad-CCM in terms of MSE by 46% and 14%, respectively.

**Figure 8:**
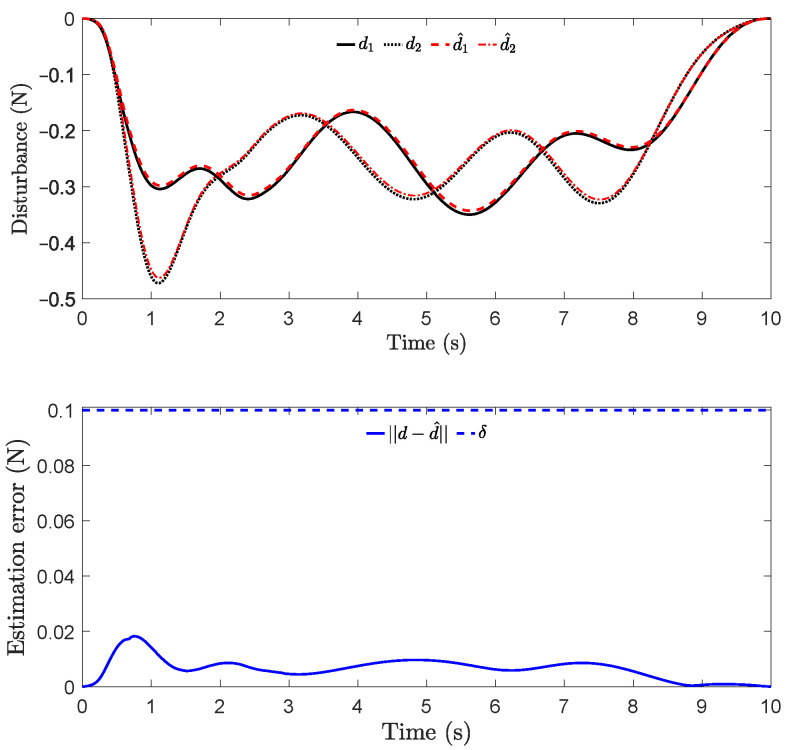
Actual and estimated disturbances (top) and the estimation error (bottom). Note that $d_{i}$ and ${\hat{d}}_{i}$ (i=1,2) represent the *i*th element of *d* (actual disturbance) and $\hat{d}$ (estimated disturbance), respectively. The blue dashed line in the bottom plot denotes the EEB used in computing the control inputs.

From Figure 6, one can see that the magnitude of $E(x^{⋆},x)$ under the RD-CCM controller decreased exponentially, and the magnitude was bounded by the curve $E\left(x^{⋆}\left(0\right),x\left(0\right)\right)e^{-2\lambda t}$ from above except at the very end when the energy is close to zero. In comparison, Ad-CCM with Γ=100 yielded similar tracking performance to DE-CCM, while the tracking performance of Ad-CCM with Γ=10 was relatively worse and characterized by larger oscillations. Additionally, from Figure 7, one can see that the control inputs generated by both of the Ad-CCM controllers have high-frequency oscillations before 3 s, which is undesired for practical deployment. Finally, Figure 8 shows the actual and estimated disturbances as well as the estimation error. One can see that the estimated disturbance is quite close to the actual one for both channels, and the EEB of 0.1 is respected throughout the simulation.

## 5. Conclusions

This paper presents a robust trajectory tracking controller with exponential convergence for uncertain nonlinear systems based on control contraction metrics (CCM) and disturbance estimation. The controller uses a disturbance estimator to estimate the pointwise value of the uncertainty with a pre-computable estimation error bound (EEB). The estimated disturbance and the EEB are then incorporated into a robust Riemannian energy condition, which guarantees exponential convergence of actual trajectories to desired ones. The efficacy of the proposed controller is validated in simulations. In particular, the proposed controller outperforms an existing adaptive CCM controller in terms of tracking performance by 2% for the aircraft example and 14% for the planar quadrotor example, while not needing to know the basis functions to parameterize the uncertainties that are needed by the adaptive CCM controller.

This paper considers only matched uncertainties, which are added to the system through the same channels as control inputs. In the future, we would like to address unmatched uncertainties that widely exist in practical systems. Additionally, we would like to experimentally validate the proposed controller on real hardware.

## Figures and Tables

**Table 1:** MSE for state trajectory tracking for the aircraft example.

	CCM	Ad-CCM	DE-CCM
MSE	$2.994\times 10^{-3}$	$1.397\times 10^{-3}$	$1.369\times 10^{-3}$

**Table 2:** MSE for state trajectory tracking for the planar quadrotor example. For Ad-CCM, only the result for Γ=100, which corresponds to better tracking performance compared to Γ=10, is included.

	CCM	Ad-CCM (Γ=100)	DE-CCM
MSE	1.175	0.738	0.634

## Data Availability

Not available.

## Appendix A

**Proof of Lemma** **4:** Hereafter, we use the notations $Z_{i}$ and $Z_{1}^{n}$ to denote the integer sets {i,i+1,i+2,⋯} and {1,2,⋯,n}, respectively. Additionally, for notation brevity, we define σ(t)≜B(x(t))d(x(t)). From (1) and (18), the prediction error dynamics are obtained as

(A1) $$\dot{\tilde{x}}\left(t\right)=-a\tilde{x}\left(t\right)+\hat{\sigma }\left(t\right)-\sigma \left(t\right),\;\tilde{x}\left(0\right)=0.$$

Note that $\hat{\sigma }\left(t\right)=0$ (and thus $\hat{d}\left(t\right)=0$ due to (20)) for any t∈[0,T) according to (19). Further considering the bound on d(t,x) in (4), we have

(A2) $$\|\hat{d}\left(t\right)-d\left(t,x\left(t\right)\right)\|\leq b_{d},\;\forall t\in \left[0,T\right).$$

We next derive the bound on $\left\|\hat{\sigma }\left(t\right)-\sigma \left(t\right)\right\|$ for t≥T. For any t∈[iT,(i+1)T) ($i\in Z_{0}$), we have

$$\tilde{x}\left(t\right)=e^{-a(t-iT)}\tilde{x}\left(iT\right)+\int_{iT}^{t}e^{-a(t-\tau )}\left(\hat{\sigma }\left(\tau \right)-\sigma \left(\tau \right)\right)d\tau .$$

Since $\tilde{x}\left(t\right)$ is continuous, the preceding equation implies

(A3) $$\begin{array}{cc} \tilde{x}\left(\left(i+1\right)T\right) & =\;e^{-aT}\tilde{x}\left(iT\right)+\;\int_{iT}^{(i+1)T}\;e^{-a((i+1)T-\tau )}d\tau \hat{\sigma }\left(iT\right)-\;\int_{iT}^{(i+1)T}\;e^{-a((i+1)T-\tau )}\sigma \left(\tau \right)d\tau \\ \; & =\;e^{-aT}\tilde{x}\left(iT\right)\;+\;\frac{1-e^{-aT}}{a}\hat{\sigma }\left(iT\right)\;-\;\int_{iT}^{(i+1)T}\;e^{-a((i+1)T-\tau )}\sigma \left(\tau \right)d\tau \\ \; & =\;-\int_{iT}^{(i+1)T}e^{-a((i+1)T-\tau )}\sigma \left(\tau \right)d\tau , \end{array}$$

where the first and last equalities are due to the estimation law (19).

Since x(t) is continuous, σ(t)(=B(x)d(x)) is also continuous given Assumption 1. Furthermore, considering that $e^{-a((i+1)T-\tau )}$ is always positive, we can apply the first mean value theorem in an element-wise manner (Note that the mean value theorem for definite integrals only holds for scalar valued functions) to (A3), which leads to

(A4) $$\begin{array}{cc} \tilde{x}\left(\left(i+1\right)T\right)= & -\int_{iT}^{(i+1)T}e^{-a((i+1)T-\tau )}d\tau [\sigma_{j}\left(\tau_{j}^{*}\right)]=-\frac{1}{a}\left(1-e^{-aT}\right)[\sigma_{j}\left(\tau_{j}^{*}\right)], \end{array}$$

for some $\tau_{j}^{*}\in \left(iT,\left(i+1\right)T\right)$ with $j\in Z_{1}^{n}$ and $i\in Z_{0}$, where $\sigma_{j}\left(t\right)$ is the *j*-th element of σ(t), and

$$[\sigma_{j}\left(\tau_{j}^{*}\right)]≜{\left[\sigma_{1}\left(\tau_{1}^{*}\right),\cdots ,\sigma_{n}\left(\tau_{n}^{*}\right)\right]}^{⊤}.$$

The estimation law (19) indicates that for any *t* in [(i+1)T,(i+2)T), we have $\hat{\sigma }\left(t\right)=-\frac{a}{e^{aT}-1}\tilde{x}\left(\left(i+1\right)T\right).$ The preceding equality and (A4) imply that for any *t* in [(i+1)T,(i+2)T) with $i\in Z_{0}$, there exist $\tau_{j}^{*}\in \left(iT,\left(i+1\right)T\right)$ ($j\in Z_{1}^{n}$) such that

(A5) $$\hat{\sigma }\left(t\right)=e^{-aT}[\sigma_{j}\left(\tau_{j}^{*}\right)].$$

Note that

(A6) $$\begin{array}{c} \|\sigma \left(t\right)\;-\;[\sigma_{j}\left(\tau_{j}^{*}\right)]\|\leq \;\sqrt{n}{\left\|\sigma \left(t\right)\;-\;[\sigma_{j}\left(\tau_{j}^{*}\right)]\right\|}_{\infty }\;\;=\;\sqrt{n}\left|\sigma_{{\bar{j}}_{t}}\left(t\right)\;-\;\sigma_{{\bar{j}}_{t}}\left(\tau_{{\bar{j}}_{t}}^{*}\right)\right|\leq \;\sqrt{n}\left\|\sigma \left(t\right)\;-\;\sigma (\tau_{{\bar{j}}_{t}}^{*})\right\|, \end{array}$$

where ${\bar{j}}_{t}=\arg\max_{j\in Z_{1}^{n}}\left|\sigma_{j}\left(t\right)-\sigma_{j}\left(\tau_{j}^{*}\right)\right|$. Similarly,

(A7) $$\begin{array}{cc} \|\left[\sigma_{j}\left(\tau_{j}^{*}\right)\right]\|\leq & \sqrt{n}{∥[\sigma_{j}\left(\tau_{j}^{*}\right)]∥}_{\infty }=\sqrt{n}\left|\sigma_{\hat{j}}\left(\tau_{\hat{j}}^{*}\right)\right|\leq \sqrt{n}\|\sigma (\tau_{\hat{j}}^{*})\|\leq \sqrt{n}b_{d}\max_{x\in X}\|B(x)\|, \end{array}$$

where $\hat{j}=\arg\max_{j\in Z_{1}^{n}}\left|\sigma_{j}\left(\tau_{j}^{*}\right)\right|$, and the last inequality is due to the fact ‖B(x)d(t,x)‖≤‖B(x)‖‖d(t,x)‖ and (4). Therefore, for any t∈[(i+1)T,(i+2)T) ($i\in Z_{0}$), we have

(A8) $$\begin{array}{cc} \left\|\sigma \left(t\right)\;-\;\hat{\sigma }\left(t\right)\|=\|\sigma \left(t\right)\;-\;e^{-aT}\left[\sigma_{j}\left(\tau_{j}^{*}\right)\right]\|\leq \right\| & \sigma \left(t\right)\;-\;[\sigma_{j}\left(\tau_{j}^{*}\right)]\|\;+\;\left(1\;-\;e^{-aT}\right)\left\|[\sigma_{j}\left(\tau_{j}^{*}\right)]\right\|\\ \leq & \|\sqrt{n}\sigma \left(t\right)\;-\;\sigma (\tau_{{\bar{j}}_{t}}^{*})\|+\;\left(1\;-\;e^{-aT}\right)\sqrt{n}b_{d}\max_{x\in X}\|B(x)\|, \end{array}$$

for some $\tau_{{\bar{j}}_{t}}^{*}\in \left(iT,\left(i+1\right)T\right)$, where the equality is due to (A5), and the last inequality is due to (A6) and (A7). The dynamics in (1) indicates that

(A9) $$\|\dot{x}\|\leq \|f(x)+B(x)u\|+\left\|B(x\right)\|\left\|d(t,x\right)\|\leq \phi ,$$

where ϕ is defined in (23). As a result, the inequality (A9) implies that

(A10) $$\begin{array}{cc} \|x\left(t\right)-x\left(\tau_{{\bar{j}}_{t}}^{*})\right\| & \leq \int_{\tau_{{\bar{j}}_{t}}^{*}}^{t}\|\dot{x}\left(\tau )\right\|d\tau \leq \int_{\tau_{{\bar{j}}_{t}}^{*}}^{t}\phi d\tau =\phi \left(t-\tau_{{\bar{j}}_{t}}^{*}\right)\leq 2\phi T, \end{array}$$

where the last inequality is due to the fact that

(A11) $$t\in \left[\left(i+1\right)T,\left(i+2\right)T\right)\mathrm{and}\tau_{{\bar{j}}_{t}}^{*}\in \left(iT,\left(i+1\right)T\right)$$

Therefore, we have

(A12) $$\begin{array}{c} ∥\sigma \left(t\right)\;-\;\sigma \left(\tau_{{\bar{j}}_{t}}^{*}\right)∥=∥B\left(x\left(t\right)\right)\left(d\left(t,x\left(t\right)\right)\;-\;d\left(\tau_{{\bar{j}}_{t}}^{*},x\left(\tau_{{\bar{j}}_{t}}^{*}\right)\right)\right)\;+\;\left(B\left(x\left(t\right)\right)\;-\;B\left(x\left(\tau_{{\bar{j}}_{t}}^{*}\right)\right)\right)d\left(\tau_{{\bar{j}}_{t}}^{*},x\left(\tau_{{\bar{j}}_{t}}^{*}\right)\right)∥\\ \leq ∥B(x(t))∥∥d\left(t,x\left(t\right)\right)\;-\;d\left(\tau_{{\bar{j}}_{t}}^{*},x\left(\tau_{{\bar{j}}_{t}}^{*}\right)\right)∥+∥B\left(x\left(t\right)\right)-B\left(x\left(\tau_{{\bar{j}}_{t}}^{*}\right)\right)∥∥d(\tau_{{\bar{j}}_{t}}^{*},x\left(\tau_{{\bar{j}}_{t}}^{*}\right))∥\\ \leq \left(L_{d}∥x\left(t\right)-x\left(\tau_{{\bar{j}}_{t}}^{*}\right)∥+l_{d}\left(t-\tau_{{\bar{j}}_{t}}^{*}\right)\right)\max_{x\in X}∥B(x)∥+L_{B}∥x\left(t\right)-x\left(\tau_{{\bar{j}}_{t}}^{*}\right)∥b_{d}\\ \leq 2T\left(\left(L_{d}\phi +l_{d}\right)\max_{x\in X}∥B(x)∥+L_{B}b_{d}\right), \end{array}$$

where the second inequality is due to Assumption 1 and the last inequality is due to (A10) and (A11). Finally, plugging (A12) into (A8) leads to

 ‖σ(t)−σ^(t)‖≤2nT(Ldϕ+ld)maxx∈XB(x)+LBbd+(1−e−aT)nbdmaxx∈XB(x)(A13)=2nT(Ldϕ+ld)+(1−e−aT)nbdmaxx∈XB(x)+2nTLBbd(A14)=α(T),

for any t≥T. From (A2) and (A14) and the relation between $\hat{\sigma }\left(t\right)$ and $\hat{d}\left(t\right)$ in (20), we arrive at (21). Considering Assumption 1 and the assumption that X and U are compact, the constants involved in the definition of α(T) in (22) are all finite. As a result, we have $\lim_{T\to 0}\alpha \left(T\right)=0$, which further indicates that $\lim_{T\to 0}\delta \left(t,T\right)=0,$ for any t≥T. The proof is complete. □
